# Study on a Mechanism of Improving MaAPX1 Protein Activity by Mutating Methionine to Lysine

**DOI:** 10.3390/antiox13070843

**Published:** 2024-07-14

**Authors:** Lu Xiao, Guoxiang Jiang, Hongmei Lai, Xiaoyan Duan, Huiling Yan, Shaoge Chen, Zexin Chen, Xuewu Duan

**Affiliations:** 1 Guangdong Provincial Key Laboratory of Applied Botany, South China Botanical Garden, Chinese Academy of Sciences, Guangzhou 510650, China; xiaolu@gdaas.cn (L.X.); gxjiang@scbg.ac.cn (G.J.); laihongmei@scbg.ac.cn (H.L.); duanxiaoyan@scbg.ac.cn (X.D.); shaogechenlin@163.com (S.C.); 2 Institute of Quality Standard and Monitoring Technology for Agro-Products of Guangdong Academy of Agricultural Sciences, Guangzhou 510640, China; 3 School of Food and Biological Engineering, Chengdu University, Chengdu 610106, China; yhl201428010715026@163.com; 4 Accurate International Biotechnology Co., Ltd., Guangzhou 510535, China; czxchase@126.com

**Keywords:** ascorbate peroxidase 1 (APX1), activity, S-nitrosylation, enzyme kinetics, molecular docking

## Abstract

Ascorbate peroxidases (APXs) are key components of the ascorbate–glytathione cycle, which plays an important role in removing excess reactive oxygen species (ROS) in plants. Herein, MaAPX1 was verified as being involved in the ripening and senescence of banana fruit, exhibiting responsiveness to the accumulation of ROS and the oxidation of proteins. Site-directed mutation was applied to explore the mechanism of MaAPX1 activity changes. We found that the 32-site cysteine (Cys, C) served as a potential S-nitrosylation site. The mutant MaAPX1^C32S^ activity was decreased significantly when Cys32 was mutated to serine (Ser, S). Intriguingly, the neighboring conserved 36-site methionine (Met, M), which is adjacent to Cys32, displayed an enzyme activity that was approximately five times higher than that of the wild-type MaAPX1 when mutated to lysine (Lys, K). Utilizing LC-MS/MS spectroscopy coupled with stopped-flow analysis showed that the enhanced MaAPX1^M36K^ activity might be due to the increased S-nitrosylation level of Cys32 and the promotion of intermediate (compound **I**, the first intermediate product of the reaction of APX with H_2_O_2_) production. Molecular docking simulations showed that the S-N bond between Cys32 and Lys36 in MaAPX1^M36K^ might have a function in protecting the thiol of Cys32 from oxidation. MaAPX1^M36K^, a promising mutant, possesses immense potential for improving the antioxidant capabilities of APX in the realm of bioengineering technology research.

## 1. Introduction

Reactive oxygen species (ROS), as inevitable byproducts of biological aerobic metabolism in plants, play a pivotal role in various physiological processes [1]. Unbalanced ROS metabolism caused by adversity or aging can lead to the oxidative damage of biological macromolecules (proteins, lipids, nucleic acids, etc.), destroy body structure and function, and, finally, lead to programmed cell death and tissue aging [2]. Plants are endowed with some ROS-scavenging mechanisms, including H_2_O_2_-metabolizing enzymes (ascorbate peroxidase (APX), thioredoxin-dependent peroxidase (PRX), superoxide dismutase (SOD), peroxidase (CAT), etc.), and nonenzymatic antioxidant systems (such as glutathione, vitamin, ubiquinone, etc.) [3]. Among these antioxidant systems, APX has a higher affinity for H_2_O_2_ content and is known to be involved in environmental stresses relating to plants, such as drought [4], salt [5], chilling [6], photo-oxidative stress [7], and high temperatures [8], as well as some physiological and developmental responses, such as seed germination [9], leaf senescence [10], root nodule development [11], lateral root formation [12], and programmed cell death [13]. On the contrary, APX deficiency leads to sensitivity to various oxidative stresses [14,15].

APX activity is regulated by multiple post-translational modifications, such as S-nitrosylation, carbonylation, glutathionylation, metal nitrosylation, and Cys oxidation and nitration [16]. The cys32 in APX is highly conserved and present (100%) in the cytoplasm of APX [17,18]. Cys32 is part of the pocket that binds to ASC (ascorbate), and its nitrosylation can promote APX activity [19]. In the case of ROS accumulation, the activity of *A. toxicaria* APX can be inhibited by carbonylation modification in seed-drying processes [20]. The oxidized cysteine in APX can be reduced by the NADPH-Trx (nicotinamide adenine dinucleotide phosphate–thioredoxin) system to restore protein activity [21], and APX activity is inhibited when the thiol group of cysteine is affected by reduced glytathione [17]. Furthermore, APX, as a typical heme protein, has an inhibitory effect on its activity when its heme subunit undergoes metal chelation [22,23]. As a pivotal antioxidant protein capable of efficiently neutralizing reactive oxygen species, augmenting the activity of APX is crucial for enhancing plant resilience against environmental stresses. To date, in addition to the documented enhancement of APX activity through S-nitrosylation modification, there is an imperative need to elucidate and establish novel methods that can potentiate the enzymatic activity of APX.

Bananas (*Musa* sp. cv. Brazil) are one of the most economically important fruits and subject to a swift process of ripening and senescence once harvested. APX is a pivotal component of the antioxidant enzyme system. Our preliminary study revealed that mutating Met36 into glutamine (Gln, Q) in MaAPX1 (Ma02_p20860.1) significantly decreased APX activity by hindering the formation of compound **I** [24]. The transformation of Met36 into various amino acids elicited diverse responses in enzyme activity. In our present study, when the Met36 in MaAPX1 was mutated into lysine (K), the enzyme activity improved, showing enzyme activity that was about five times higher than that of the wild-type MaAPX1. To elucidate the underlying mechanism of this activity enhancement in MaAPX1^M36K^, we employed a multi-faceted approach, including LC-MS/MS (liquid chromatography–mass spectrometry), spectral analysis, and molecular docking. MaAPX1^M36K^, as a new mutant material, offers immense potential in augmenting the antioxidant function of APX in the realm of bioengineering technology research.

## 2. Materials and Methods

### 2.1. Plant Materials and Treatments

Bananas (*Musa* sp.cv. Brazil) with a maturity of 70–80% were harvested from Nansha District, Guangzhou, Guangdong Province, China, and transported to a laboratory within 2 h. Fruits with uniform size and color were selected and soaked in 0.1% Sportak^®^ (Prochloraz, Bayer, Leverkusen, Germany) for 3 min and then allowed to air-dry.

Three treatments were set up in this experiment, including a control, an ethylene treatment, and a 1-MCP treatment. Sixty dried bananas were placed in an 80 L sealed plastic box to ensure air circulation inside. For the ethylene treatment group, the sealed box was filled with 40 mL of ethylene; for the 1-MCP (1-Methylcyclopropene, ethylene receptor inhibitor) treatment group, 1-MCP was placed in the sealed box to achieve a final concentration of 1 ppm. All the covers of the sealed boxes were removed for ventilation after 24 h. Each treatment had 3 replicates. Based on the hardness and color changes during banana storage, samples were taken to determine the relevant physiological indicators. Partial samples were stored in a −80 °C refrigerator for RNA extraction.

### 2.2. Measurement of Physiological Parameters

Banana peel color was measured with a Konica Minolta CR-400 colorimeter (Konica Minolta Co., Ltd., Tokyo, Japan) in the CIEL*a*b mode. L* indicated the lightness ranging from black to white, and hue angle (h*) referred to a color wheel, which was red at an angle of 0°, yellow at 90°, green at 180°, and blue at 270°, in accordance with the method outlined by McGuire [25].

The H_2_O_2_ content and protein carbonylation level in the banana peels were determined using a hydrogen peroxide kit and protein carbonyl content kit (Suzhou Keming Biotechnology Co., Ltd., Suzhou, China), respectively. The content was expressed as μmol g^−1^ FW.

The steps taken for total APX enzyme extraction and activity measurement were as follows: One point five grams of fresh banana peel was ground in liquid nitrogen and dissolved in 3 mL of pre-cooled 50 mM potassium phosphate buffer (pH7.0) to extract APX for 10 min. The mixture was centrifuged at 4 °C at 10,000 rpm, and the supernatant was collected to measure the enzyme activity. Reaction system: 1 mL 50 mM potassium phosphate buffer (pH 7.0), 0.4 mL of 0.3 mM EDTA-Na_2_, and 1 mL of 0.9 mM VC were mixed, and 0.1 mL of enzyme solution was added to initiate the reaction. We recorded the changes in the OD value of the reaction solution at 290 nm for 2 min and repeated this process three times. APX activity (μmol VC mg^−1^ min^−1^ FW) = ΔA × Vt × v/(FW × Vs × 2.8 × t); ‘Vt’—total volume of enzyme solution, mL; ‘FW’—fresh weight of the sample, mg; ‘Vs’—volume of enzyme solution taken during measurement, mL); ‘v’—volume of reaction solution, mL; ‘ΔA’— the absorbance value changes of OD_290_ within 60 s; ‘t’—Reaction time, min; and ‘2.8’—the millimolar extinction coefficient of VC.

### 2.3. Plasmid Construction and Gene Expression

The coding sequences of *MaAPX1* were PCR-amplified from banana complementary DNA. Total RNA was extracted from banana peel tissues using the hot borate method [26]. DNA-free RNA was used as the template for reverse-transcription PCR. The gene-specific primers used for gene cloning and gene expression are listed in Table S2. The PCR products were sub-cloned into the pMD20-T vector (TaKaRa) and then transformed into *E. coli* DH5α (TaKaRa) in accordance with the manufacturer’s protocol. The sequences were verified via further cloning and resequencing. The resulting sequences were deposited in Banana Genome Hub https://banana-genome-hub.southgreen.fr/ (accessed on 9 July 2024) with the accession number *GSMUA_Achr5P07280_001 (MaAPX1)*. The alignments were carried out using ClustalX (version 1.83). The qRT-PCR reactions for MaAPX1 were carried out using the ABI 7500 Real-Time PCR System (Applied Biosystems, Carlsbad, CA, USA) with SYBR Green Real-Time PCR Master Mix (TOYOBO Co., Ltd., Osaka, Japan). The conditions of the reactions were set with reference to the work by Xiao et al. [27] with the reference gene *MaActin-3* [28]. Three independent biological replicate experiments were performed in this analysis.

### 2.4. Site-Directed Mutagenesis of Cys32 and Met36 Residues

The following site-directed mutagenesis of Cys32 or Met36 was performed: mutations of Cys32 into serine (S) and Met36 into lysine (K) were carried out as described previously [24]. The full-length MaAPX1 mutants were sub-cloned into DH5α and verified through DNA sequencing. The oligonucleotide sequences of responding mutation sites in MaAPX1 for RT-PCR are shown in Table S2.

### 2.5. Purification of Recombinant Proteins

The sequences encoding MaAPX1 or MaAPX1 mutants were inserted into pET-28a (+) vector (Novagen, Irene, South Africa). The His-fusion proteins were induced and expressed in *E.coli* BL21 (DE3) strain (TransGen Biotech, Beijing, China). The recombinant proteins were then purified with nickel-nitrilotriacetic acid agarose (Qiagen, Hilden, Germany), following the manufacturer’s instructions. The results of SDS-PAGE of purified expressed recombinant MaAPX1-His and the mutants from *E. coli* BL21 are shown in Figure S2.

### 2.6. Enzymatic Activity of MaAPXs Measurement

MaAPX activity was measured according to the method reported by Yang et al. [19], with some modifications. The protein concentrations of MaAPXs were adjusted to 0.1 mg/mL with 50 mM sodium phosphate (pH 7.0). As typically carried out, 500 μL of the reconstitution products was used for the assay by adding sodium ascorbate and hydrogen peroxide to final concentrations of 0.45 mM and 1 Mm, respectively. After initiating the reaction by adding hydrogen peroxide, the samples were immediately analyzed with UVmini-1240 by measuring the absorbance at A290 every 15 s for 2 min. APX activity (μM AsA min^−1^ μg^−1^) =ΔA × 1000 × 1000/(C × Vs × 2.8 × t); ‘C’—concentration of the sample, mg/mL; ‘Vs’—volume of enzyme solution, μL; ‘ΔA’— the absorbance value changes at OD290 within 1 min; ‘t’—reaction time, min; and ‘2.8’—the millimolar extinction coefficient.

To investigate the effect of in vitro S-nitrosation on protein activity, the solvent of purified MaAPX1-His was replaced with HEN buffer (250 mM Hepes, pH 7.7; 1 mM EDTA-Na_2_; 0.2 mM neocuproine) using an ultrafiltration centrifuge tube. Then, the protein was added to a final concentration of 750 μM GSNO (Sigma Aldrich, St. Louis, MO, USA, Cat no.: N4148) and incubated in a dark room. After one hour, the solvent of the protein was replaced with 50 mM sodium phosphate (pH 7.0), and then the protein activity was measured.

### 2.7. Mass-Spectrometric Analysis of S-Nitrosylation Level of Proteins

Mass-spectrometric analysis of S-nitrosylation level of proteins was conducted by Shenzhen Wininnovate Biotechnology Limited Company (Shenzhen, China).

Protein digestion. A total of 50 μg of purified MaAPX1-His was denatured with 8 M urea and then diluted to 1 M urea with 50 mM NH_4_HCO_3_. The protein dilution material was digested with trypsin (1:50 enzyme/protein ratio) at 37 °C for 16–18 h and acidified with 1% TFA (Trifluoroacetic Acid). The peptides were lyophilized.

Nano LC-ESI-MS/MS analysis. The lyophilized peptides were re-suspended in 2% acetonitrile containing 0.1% formic acid, 4 μL aliquots of which were loaded into a ChromXP C18 (3 μm, 120 Å) trap column. Online chromatography separation was performed using the Ekspert nanoLC 415 system (SCIEX, Concord, ON, Canada). The trapping-and-desalting procedure was carried out at a flow rate of 4 μL/min for 5 min with 100% solvent A (water/acetonitrile/formic acid (98/2/0.1%). Then, an elution gradient of 8–38% solvent B (2/98/0.1%) in 40 min was used on an analytical column (75 μm × 15 cm C18-3 μm 120 Å, ChromXP, Eksigent, Dublin, CA, USA). IDA (information-dependent acquisition) mass spectrum techniques were used to acquire tandem MS data using a Triple TOF 6600 tandem mass spectrometer (Sciex, Concord, ON, Canada) fitted with a Nano spray III ion source. Data were acquired using an ion spray voltage of 2.4 kV, curtain gas of 35 PSI, nebulizer gas of 12 PSI, and an interface heater temperature of 150 °C. The MS was operated with TOF-MS scans. For IDA, survey scans were acquired in 250 ms, and up to 40 product ion scans (50 ms) were collected if a threshold of 260 cps with a charge state of 2–4 was exceeded. The rolling collision energy setting was applied to all precursor ions for collision-induced dissociation. Dynamic exclusion was set for 16 s.

Database searching. The MS/MS data were analyzed for protein modification (Carboxamidomethylation (+57.02) of cysteine, oxidation (+15.99) of methionine, acetylation (+42.01) of Protein N-term (deamidation (+0.98) of asparagine and S-nitrosylation (+28.99) of cysteine were allowed as variable modifications), and quantification using PEAKS Studio version 8.5 (BSI, San Jose, CA, USA) against protein sequence. The threshold used for peptide identification was a peptide for which −10lgP > 20 and that had a PTM A score > 500. Mass accuracy of ±20 ppm was used for precursor ions, and 0.5 Da was used for product ions. Enzyme specificity was limited to trypsin, with at least one tryptic (K- or R-containing) terminus required per peptide and up to two miscleavages being allowed. Cysteine carboxamidomethylation was specified as a static modification; oxidations of methionine residue and the appropriate PTM were allowed as variable modifications.

### 2.8. Spectroscopic Analysis and Stopped-Flow Analysis

According to the method employed by Hugo et al. [29], UV-visible spectra of Asc-free MaAPX1 and mutant MaAPX1^M36K^ (1 μg/μL) samples were recorded at 25 °C in the presence or absence of an equimolar H_2_O_2_ concentration in potassium phosphate buffer (50 mM, pH 7.0).

Rapid-acquisition spectra and absorbance time courses were obtained following the recording of the Asc-free MaAPX1 and mutant MaAPX1^M36K^ enzymes before and after H_2_O_2_ addition by mixing equal amounts (2 μg/μL each) in potassium phosphate buffer (50 mM, pH 7.0) containing diethylenetriaminepentaacetic acid (DTPA) (0.1 mM) at 10 °C, reaching a final concentration of 1 μg/μL of reagents. Absorbance was recorded with an Applied Photophysics 9CD stopped-flow spectrofluorometer equipped with a rapid-scanning diode array. Formation of compound **I** species followed, occurring at a Soret peak of 409 nm for MaAPX1 and 408 nm for MaAPX1^M36K^.

### 2.9. Molecular Docking Experiment

The 3D structures of MaAPX1 and its mutants (MaAPX1^M36K^) were generated by using Swiss-Model, using the crystal structure of soybean APX (PDB: 1V0H, 84.53% sequence identity) as an initial template [30]. Models of ligands (heme) were constructed using eLBOW, Phoenix, by using smile strings of ligands via the AM1/QM optimization method. The model of MaAPX1 and ligands were prepared using Autodock tool. In this case, Cys32 and Met36 (or Lys36) were set as flexible residues.

### 2.10. Statistical Analysis

Data were expressed as the means ± SE (standard error) of three biological replicates. Differences among different treatments were compared using SPSS version 7.5 (SPSS, Inc., Chicago, IL, USA). Different letters in each figure indicate significant differences (*p* < 0.05) between different treatments at the same storage time.

## 3. Results

### 3.1. Ripening Characteristics and Redox Status of Harvested Banana Fruit during Ripening and Senescence

**Figure 1:**
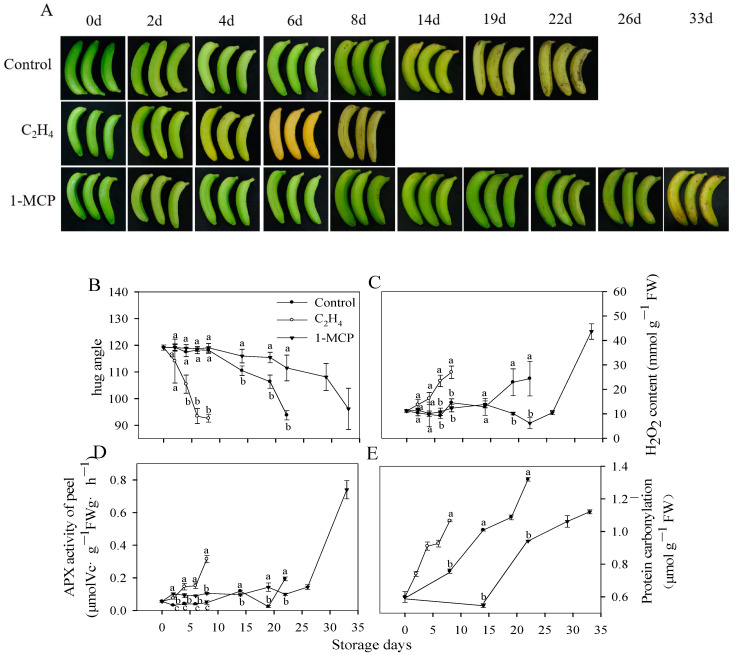
Ripening characteristics and redox statuses of harvested bananas during ripening and senescence. Changes in the ripening of the banana peels with different treatments (**A**), hug angle (**B**), H_2_O_2_ content (**C**), APX activity (**D**), and protein carbonylation (**E**). Each data point represents a mean ± standard error (*n* = 3). The values with different letters refer to each sampling from the same storage time and are significantly different (*p* < 0.05).

Bananas are a typical climacteric fruit, characterized by a pre-climacteric phase followed by a respiration peak, subsequently becoming soft and yellow. As shown in Figure 1A,B, the control fruits underwent a yellowing process after 14 days; in contrast, the ethylene (C_2_H_4_)-treated fruits exhibited yellowing on the 4nd day, and the 1-MCP treated fruits took 33 days to turn yellow. The control fruits experienced a significant reduction in hug angle, declining from 119.1 to 110.5 on the 14nd day, and this decline was accompanied with yellowing. The emergence of conspicuous spots and browning of the peel occurred on the 22nd day, with a decreased hug angle of 93.8. This indicated that the fruit ripened and underwent greater senescence as the storage time increased. Ethylene promoted yellowing of the banana peel, and the hug angle of the banana peel decreased to 105.4 on the fourth day; spots appeared on the 8nd day, with a decrease in the hug angle amounting to 92.6, which is much lower than that for the control group (*p* < 0.05). In contrast, for the 1-MCP-treated fruit, the hug angle decreased to 108.1 after 26 days of storage, and peel browning occurred after 33 days of storage. These results indicate that ethylene promoted the ripening and senescence of bananas, while 1-MCP delayed ripening and senescence.

H_2_O_2_ is a by-product produced by organisms during aerobic respiration [31]. A low concentration of H_2_O_2_ acts as an important signal, while a high concentration of H_2_O_2_ causes oxidative damage to biological macromolecules. Figure 1C shows an accumulation of H_2_O_2_ in banana peels along with different ripening stages. C_2_H_4_ treatment accelerated the accumulation of H_2_O_2_, while 1-MCP treatment delayed the accumulation of H_2_O_2_. Figure 1D shows the changes in APX activity in the banana peels, which are basically consistent with the accumulation of H_2_O_2_. APX activity in the C_2_H_4_- and 1-MCP-treated bananas increased with the accumulation of H_2_O_2_ during storage, and there was a strong correlation between APX activity and H_2_O_2_ content during fruit ripening and senescence (*p* < 0.05) (Table S1). These results indicate that APX might be a pivotal enzyme, responsible for the elimination of H_2_O_2_ during banana ripening and senescence.

Most biological macromolecules (nucleic acids, proteins, lipids) in living organisms are the target substances for ROS oxidation, and these biological macromolecules are prone to structural and functional damage and accelerate the process of aging after oxidation [32]. Carbonylation modification occurs after proteins are oxidized, and it is accompanied by the formation of sulfonic acid and sulfone substances, which have a great impact on the function of a protein [33]. Protein carbonylation levels are commonly used to evaluate the degree of protein oxidation. In the present study, protein carbonylation levels increased gradually as the storage time increased (Figure 1E), and it was observed that the C_2_H_4_ and 1-MCP treatments played roles in promoting and inhibiting protein carbonylation, respectively. These findings underscore the significance of ROS accumulation in triggering oxidative damage to biological macromolecules, thereby serving as a crucial factor in accelerating the senescence of fruits.

### 3.2. Expression Profiles of MaAPX1 Gene during Banana Ripening and Senescence

**Figure 2:**
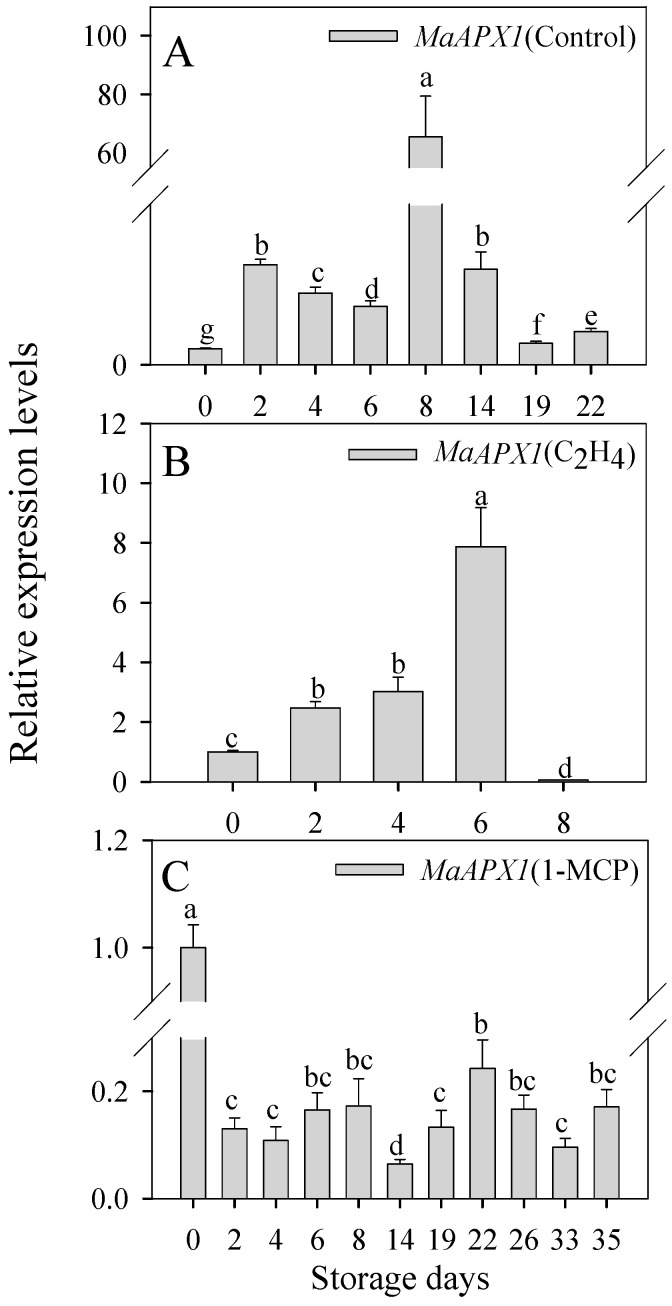
Changes in the relative expression of MaAPX1 in different treatments. Control (**A**), C_2_H_4_ treatment (**B**), and 1-MCP treatment (**C**). The relative gene expression levels of *MaAPX1* on different storage days, wherein storage day 0 was chosen as the reference, and the gene expression level on day 0 was set to 1. Each datapoint represents a mean ± standard error (*n* = 3). The values with different letters (a, b, c, etc.) in each group indicate that each sample taken at different storage times is significantly different (*p* < 0.05).

To further verify the function of MaAPX1 in banana ripening and senescence, we conducted a comprehensive analysis of *MaAPX1* gene expression in banana peels during storage (Figure 2). The relative expression level of the MaAPX1 gene, under both the control and C_2_H_4_ treatments, displayed a trend of upregulation followed by a subsequent downregulation trend as storage progressed (Figure 2A,B). In stark contrast, the MaAPX1 expression in the bananas treated with 1-MCP showed consistent downregulation throughout the entire storage phase (Figure 2C), indicating that MaAPX1 likely played a pivotal role in orchestrating the regulatory mechanisms governing fruit ripening and senescence.

### 3.3. The MaAPX1 Activity Is Promoted by S-Nitrosylation of Cys32

**Figure 3:**
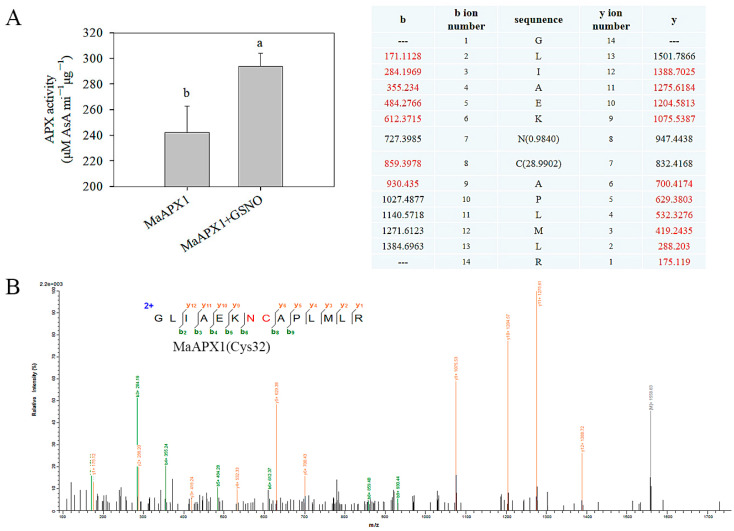
S-nitrosylation of MaAPX1-His recombinant protein. GSNO (S-Nitrosoglutathione, NO doner) enhances the enzymatic activity of MaAPX1-His recombinant protein. Purified MaAPX1-His recombinant protein was treated with GSNO for 1 h and then assayed for its enzymatic activities (**A**). Mass-spectrometric analysis of the tryptic fragments of GSNO-untreated MaAPX1-His recombinant protein, in which Cys32 was identified as an S-nitrosylated residue (the mass charge ratio (*m*/*z*) of Cys in the mass spectrum was 103.01, and it increased (+28.99) to 132.00 after S-nitrosylation). The fragment ions y5 and y6 were not detected due to their low SNRs (signal-to-noise ratios). The occurrence of S-nitrosylation in C and deamidation in N led to an increase in *m*/*z* for y7, y8, y9, and y10 (+28.99 + 0.98) that indicated we could still be sure that C was S-nitrosylated and N was deamidated. The associated mass ions are given in red in the table. (**B**). Each datapoint represents a mean ± standard error (*n = 3*). The values with different letters indicate that each sampling from different treatment groups is significantly different (*p* < 0.05).

The function of cAPX (cytoplasmic APX) is regulated by multiple PTMs (post-translational modifications), among which S-nitrosylation is known to play an important role in regulating cAPX functions. APX1 was identified as a putative S-nitrosylation protein through nitrosoproteomic studies [34,35]. It has been reported that AtAPX1 enzymatic activity can be enhanced by S-nitrosylation at Cys32 [19]. In our present study, the activity of MaAPX1 recombinant protein improved by nearly 20% after treatment with the NO doner GSNO (Figure 3A), and we identified Cys32 as the S-nitrosylated residue in MaAPX1 recombinant protein via LC-MS/MS (Figure 3B), indicating that MaAPX1 is one of the substrates of S-nitrosylation whose enzymatic activity is positively regulated by S-nitrosylation.

### 3.4. Mutant MaAPX1^M36K^ Activity Might Be Enhanced by Improving the S-Nitrosylation Level of Cys32

If continuous fragmentation of y5 and y6 occurs, it can be clearly seen that the corresponding *m*/*z* of y5 ion will increase by 28.99 after S-nitrosylation in Cys, and the corresponding y6 ion *m*/*z* will increase by 0.98 after deamidation in Asn (N).

**Figure 4:**
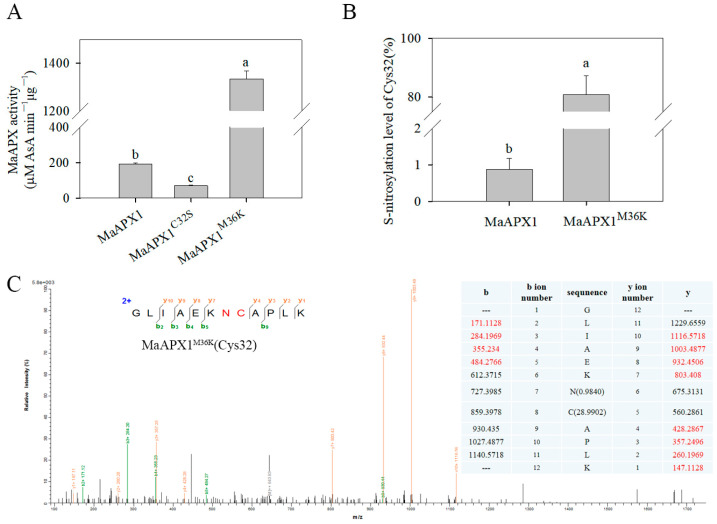
Analysis of the enzymatic activity of MaAPX1-His and the mutants’ recombinant proteins via spectrophotometry (**A**). S-nitrosylation levels of Cys32 in MaAPX1-His and MaAPX1^M36K^-His recombinant proteins (**B**). Mass-spectrometric analysis of the tryptic fragments of GSNO-untreated MaAPX1^M36K^-His recombinant protein, in which Cys32 was identified as an S-nitrosylated residue (**C**). Each datapoint represents a mean ± standard error (*n* = 3). The values with different letters indicate that each sampling from different treatment groups is significantly different (*p* < 0.05).

There are two cysteine residues in MaAPX1, namely, Cys32 and Cys167, of which Cys32 is the candidate S-nitrosylation residue in MaAPX1 identified via LC-MS/MS (Figure 3B) combined with the prediction made using GPS-GNO software [36]. The Cys32 and Met36 in APXs are highly conserved in various species (Figure S1). There was a typical active site, APLM_36_LRLAWHSA, at the N-terminus end, accompanied by a heme ligand of DIVALSGGHTL in MaAPX1, signifying the pivotal position of Met36 in the enzymatic core. As shown in Figure 4A, the substitution of Cys32 with a Ser residue (termed MaAPX1^C32S^) led to a more than 60% reduction in MaAPX1 activity, while the substitution of Met36 with Lys (termed MaAPX1^M36K^) led to an increase in enzymatic activity that was about five times higher than that for MaAPX1. These results indicate that Cys32 and Met36 are important for the function of MaAPX1. In addition, the relative S-nitrosylation level of Cys32 in the mutant MaAPX1^M36K^ recombinant protein was much higher than that of wild MaAPX1, as determined using LC-MS/MS, indicating that MaAPX1^M36K^ was more prone to S-nitrosylation, and MaAPX1^M36K^ might be S-nitrosylated during the process of prokaryotic expression or protein purification (Figure 4B). Combined with the improvement of enzymatic activity as well as the S-nitrosylation level on Cys32 for MaAPX1^M36K^, it can be inferred that S-nitrosylation at Cys32 may play a pivotal role in promoting the activity of MaAPX1^M36K^.

### 3.5. Visible Spectrum and Stopped-Flow Analysis of MaAPX1 and Mutant MaAPX1^M36K^ Reaction with H_2_O_2_

**Figure 5:**
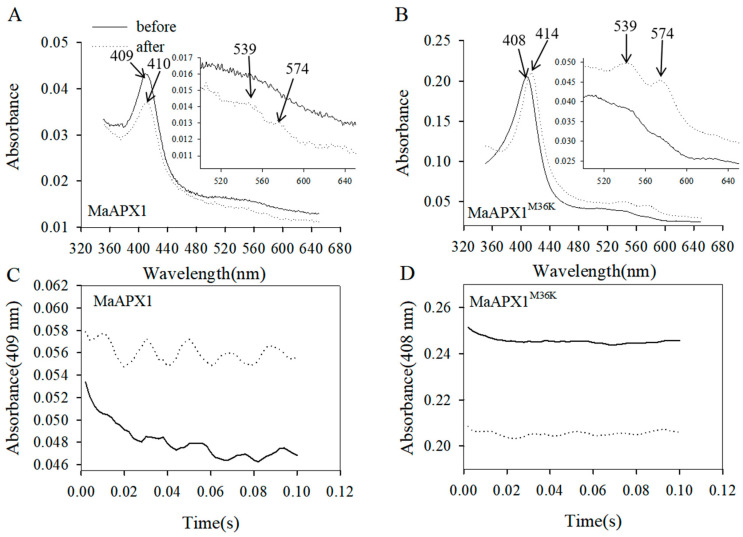
Spectroscopic analysis of the MaAPX1-His and MaAPX1^M36K^-His recombinant proteins compound **I** and compound **I**-like intermediates. Absorption spectra of MaAPX1-His (2 μM) (**A**) and MaAPX1^M36K^-His (2 μM) (**B**) before (solid line) and after (dashed line) equimolar H_2_O_2_ addition (2 μM). Spectra from 500 to 650 nm for MaAPX1-His and MaAPX1^M36K^-His. Arrows indicate the Soret peak of the resting enzyme before and after H_2_O_2_ reaction. Spectral shifts of MaAPX1^M36K^-His are indicative of compound-**I**-like formation. MaAPX1-His (5 μM) (**C**) or MaAPX1^M36K^-His (5 μM) (**D**) was mixed with H_2_O_2_ (dashed line) or left alone (solid line) (5 μM) at 10 °C. The decrease in the absorbance at the Soret peak is indicative of compound **I** generation.

In our previous study, we verified that the enzymatic activity of MaAPX1 is related to the formation of compound **I** (APX(Fe^3+^)R + H_2_O_2_ → APX(Fe^4+^ = O)R^•^ (compound **I**) + H_2_O, R, either the heme ring or an amino acid side chain) [24]. For further exploration of the mechanism of MaAPX1^M36K^ activity improvement, optical spectra of MaAPX1 and the mutant MaAPX1^M36K^ (from 350 to 650 nm) were recorded before and after the addition of an equimolar concentration of H_2_O_2_ to assay the spectral shifts associated with the formation of a compound-**I**-like product (Figure 5A,B). In the resting state, MaAPX1 and the mutant MaAPX1^M36K^ exhibited different Soret peaks at 409 nm and 408 nm (Figure 5A,B), respectively, which indicated that there was some structural perturbation in the heme microenvironment as the result of amino acid replacement [29]. As shown in Figure 5A, the Soret peak of MaAPX1 was shifted from the resting state of 409 nm to 410 nm after reaction with H_2_O_2_, with the declined two humps situated at 539 and 574 nm. The Soret peak of MaAPX1^M36K^ shifted from 408 nm to 414 nm after reaction with H_2_O_2_, with two rising humps at 539 and 574 nm (Figure 5B). Spectral shifts are indicative of compound-**I**-like formation.

Given the rapid generation of the compound-**I**-like product, rapid kinetics analysis via the stopped-flow method at 10 °C was performed to visualize the classical compound **I** in MaAPX1 and the mutant MaAPX1^M36K^. The maximal absorption (Soret peak) in the ferric state of heme peroxidases dropped in intensity when the enzyme reacted with H_2_O_2_ to generate compound **I**; a decrease in absorbance at the Soret peak is expected after H_2_O_2_ reaction [37,38,39]. In our present study, a swift drop in absorbance for MaAPX1^M36K^ (Soret peak at 408 nm) was observed when it was mixed with H_2_O_2_, indicating the formation of the Fe-^IV = O^-P^•+^ radical (P^•+^, porphyrin-centered radical) of compound **I** (Figure 5D). Contrastingly, there was no decrease in absorbance for MaAPX1 (Figure 5C), indicating that the improvement in MaAPX1^M36K^ activity might also stem from the expedited generation of compound **I**.

Furthermore, the mutant MaAPX1^M36K^ exhibited increased absorption in the Soret region with high spin state variation, indicating that the activity of iron increased and that the iron became more prone to engaging in reactions with the other substance. Conversely, a reduced spin state exhibited greater stability and a diminished tendency to interact with other substances. This underscores the pivotal role of the spin state in compound I, which significantly impacts the subsequent reaction of compound **I** (or compound **II**, APX (Fe^4+^ = O) R^•^ + ASA → APX (Fe^4+^ = O)R (compound **II**) + monodehydroascorbate) with ascorbate. Accordingly, the elevated iron spin in compound **I** coupled with the swift formation rate of compound **I** emerge as critical determinants of the elevated enzymatic activity exhibited by MaAPX1^M36K^.

### 3.6. Molecular Docking Study of MaAPX1

**Figure 6:**
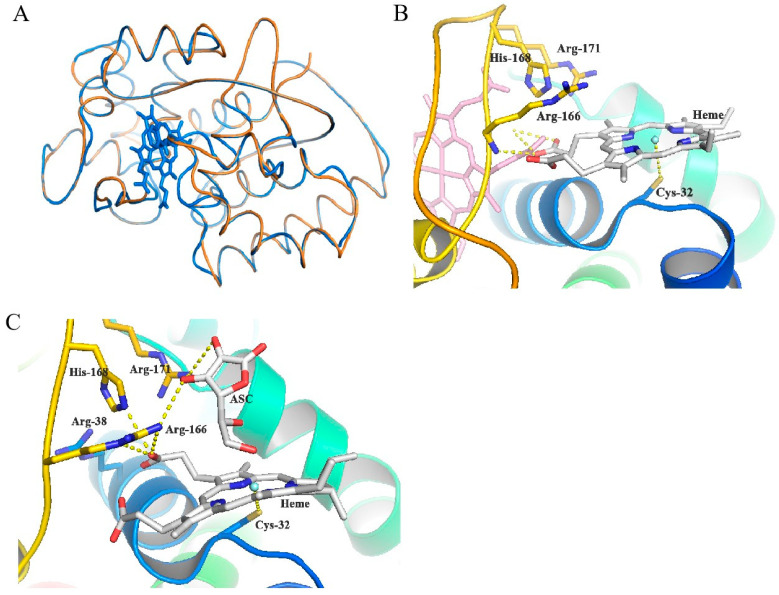
Molecular docking study of MaAPX1. Structure of predicted model (orange) and template (PDB: 1V0H) (**A**). Docking position of heme at the Cys32 binding site of MaAPX1, superimposed with heme at the heme binding site (pink) (**B**). Docking position of heme and ascorbic acid at the Cys32 binding site of MaAPX1 (**C**).

The pocket surrounding Cys32 emerges as a vital heme-binding site, crucial in enhancing the functionality of ascorbate peroxidase. Based on the high sequence identity between MaAPX1 and the soybean APX, the predicted model (depicted in orange) was generally overlayed with the template model (depicted in blue), with minor variations observed in the flexible loop’s structural conformation (Figure 6A). The alignment between MaAPX1 and its mutant variant, MaAPX1^M36K^, exhibits a striking 100% sequence identity. This high degree of conservation suggests that the mutant MaAPX1^M36K^ can comfortably integrate into the overall protein structure without perturbing its essential components, including the area around the Met36 residue (Figure S3A,B). Furthermore, Cys32 is deemed an indispensable amino acid residue in terms of the catalytic activity of cAPX, and the pocket it anchors further underlines its crucial contribution to APX activity, highlighting the significance of this region in the enzyme’s overall functionality. Watanabe et al. [40] reported that the Cys32 was the core of the heme-binding site and catalytic center in human peroxiredoxin (Prx). Therefore, it can be hypothesized that Cys32 plays an important role in ligand recognition or even catalytic activity. As depicted in Figure 6B, Cys32 was observed to be a promising site for heme recognition or binding. It may either identify heme and facilitate its transfer to the core binding site or bind to heme firmly and display catalytic prowess in ascorbic acid metabolism. In docking simulations focused on the heme’s central binding site, expanding the grid range indicated the emergence of a secondary binding locale surrounding Cys32, further validating its potential for heme interaction. This is consistent with the reduction in the enzymatic activity of MaAPX1^C32S^ (Figure 4A). Within this prospective binding locale, Cys32 orchestrates the formation of transition metal thiolate complexes with heme’s iron, effectively supplanting histidine as the heme’s binding nucleus. Moreover, three polar or positively charged residues, Arg166 (Arginine), His168 (histidine), and Arg171, were located on the opposite side of the heme molecule, whose positively charged region can act as a proton supplier. As shown in Figure 6C, the docking positions of both heme and ascorbic acid also showed that, when attached to the heme molecule, the two hydroxyl groups from ascorbic acid were capable of interacting with Arg166 and Arg171. The geometry of the Cys32 pocket fulfills the condition as an APX catalytic site.

### 3.7. The Lys36 Residue of MaAPX1^M36K^ Might Induce S-Nitrosylation of Cys32 via S-N Bonds to Enhance Enzymatic Activity

**Figure 7:**
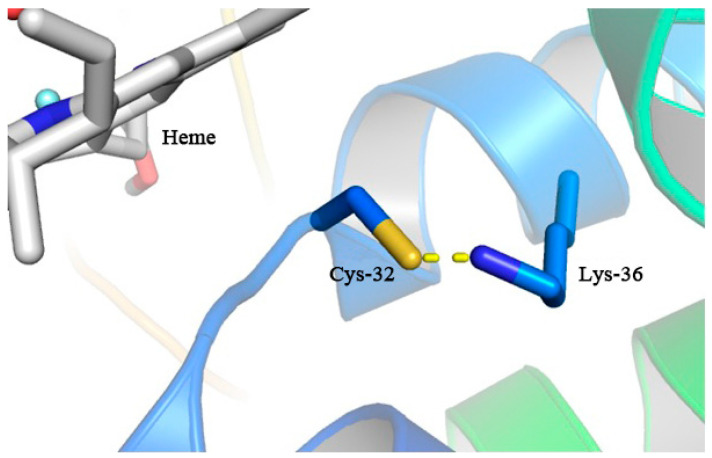
Docking of heme with APX1^M36K^, in which an interaction between Cys32 and Lys36 can be observed.

We have verified that the occurrence of S-nitroslysation at the thiol group of Cys32 significantly enhanced the activity of MaAPX1. However, unfortunately, in molecular docking simulations, the nitroso group positioned on the cysteine side chain cannot be explicitly modeled. Nonetheless, the reversibility of S-nitroslysation through de-nitrosylation processes in the presence of metal ions and under photolytic conditions ensures that the S-nitroslysation at Cys32 does not impede heme binding. As depicted in Figure 7, during the docking simulations in mutant MaAPX1^M36K^, the unrestricted torsion of Cys32 and Lys36 led to conformational alterations in Cys32, subsequently forming an S-N bond with Lys36, with the heme molecule binding to a place to that for MaAPX1. While the precise role of this S-N bond remains elusive, we postulate that Lys36 may induce the S-nitroslysation of Cys32, thereby shielding the thiol group from potential oxidative reactions and contributing to an increase in enzymatic activity.

**Figure 8:**
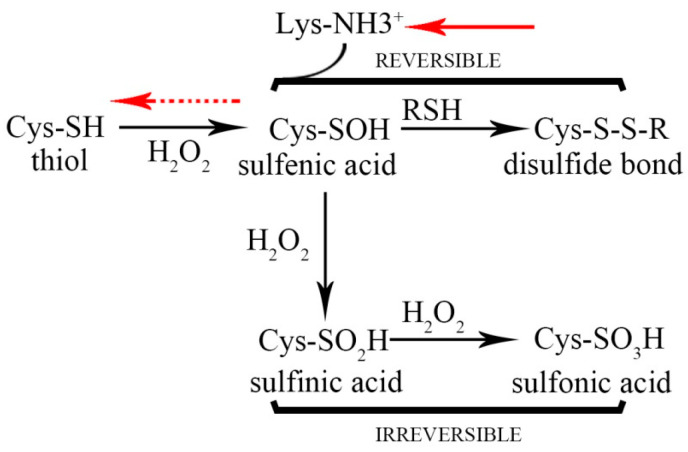
Reversible cysteine oxidation in hydrogen peroxide.

Furthermore, it has been reported that the oxidation of Cys-SH can be divided into reversible oxidation and irreversible oxidation [41]. Figure 8 shows reversible cysteine oxidation taking place in hydrogen peroxide. In the case of irreversible oxidation, the thiol group undergoes oxidation to form R-SO_3_H due to persistent exposure to H_2_O_2_ without the presence of a deoxidizer. Conversely, in the reversible oxidation pathway, Lys36 in the mutant MaAPX1^M36K^ variant, possessing a positive charge and situated in close proximity to Cys32, may serve as a source of protons, functioning as a deoxidizer for R-SOH (depicted by the red dotted arrow). This prompt reduction of R-SOH back to R-SH ensures the timely protection of Cys32 from undergoing irreversible oxidation.

After being exposed to H_2_O_2_, thiol groups of Cys are initially oxidized into a sulfenic acid form, which is highly unstable, and readily react with another proximal thiol to form adisulfide bonds. In a cellular environment, disulfide bonds can be reduced to the thiol form by the thioredoxinor the GSH/glutaredoxin systems (red solid arrow). In the presence of more H_2_O_2_, sulfenic acid can be further oxidized to sulfinic or sulfonic acid, which, in general, is an irreversible modification that cannot be repaired within cells. 

## 4. Conclusions

APX, a pivotal constituent of the ascorbic acid–glutathione cycle, holds significant importance in governing H_2_O_2_ metabolism and stands as a chief modulator of cellular redox equilibrium in plants. In the present study, MaAPX1 was shown to be involved in the process of banana ripening and senescence. The Met36 in MaAPX1, which is close to Cys32, is a crucial residue and may act as a switch regulating the enzymatic activity of MaAPX1. Intriguingly, we observed a remarkable enhancement in enzymatic activity when Met36 was substituted with Lys. Drawing upon the insights gained from LC-MS/MS, spectroscopy, stopped-flow analysis, and the comprehensive molecular docking study, we attribute the augmentation of MaAPX1^M36K^ activity to multiple factors. These include the elevation in the S-nitrosylation levels of Cys32, the accelerated formation of high-spin iron intermediates (compound **I**), and the plausible establishment of a S-N bond between Cys32 and Lys36 in MaAPX1^M36K^. This bond formation safeguards the thiol group of Cys32 from oxidation, thereby bolstering the enzymatic efficacy of MaAPX1. Emerging as a novel mutant material with the potential to enhance protein activity, MaAPX1^M36K^ presents a valuable resource for further inquiries into the functional aspects of APX and its potential applications in the realm of biotechnology.

## 5. Patents

Xuewu Duan, Lu Xiao, Guoxiang Jiang, Huiling Yan, Zhiwei Li, Jing Zeng, and Xiaochun Ding. Ascorbate peroxidase mutant MaAPX^M36K^ and application thereof. USA patent: US 11, 149, 257 B2, 2021-10-19.

## Data Availability

No new data were created.

## Supplementary Materials

The following supporting information can be downloaded at https://www.mdpi.com/article/10.3390/antiox13070843/s1: Figure S1. Sequence alignment of APX proteins from various species. Comparison of the partial sequences of MaAPX1 with APX proteins of other plant species. The Cys32 and Met36 in MaAPX1 are highlighted by arrows. AtAPX1, NP_001030991.2 ascorbate peroxidase 1 [*Arabidopsis thaliana*]; NaAPX2, XP_019250347.1 PREDICTED: L-ascorbate peroxidase 2, cytosolic [*Nicotiana attenuata*]; OsAPX2, XP_015646556.1 L-ascorbate peroxidase 2, cytosolic [*Oryza sativa* Japonica Group]; SlAPX2, NP_001318094.1 L-ascorbate peroxidase 2, cytosolic [*Solanum lycopersicum*]; CpAPX2, XP_021911667.1 L-ascorbate peroxidase 2, cytosolic [*Carica papaya*]; ZaAPX, AAC08576.1 ascorbate peroxidase [*Zantedeschia aethiopica*]. Figure S2. SDS-PAGE of purified expressed recombinant MaAPX1-His, MaAPX1^M36K^-His and MaAPX1^C32S^-His from *E. coli* BL21. Figure S3. Structure of MaAPX1 predicted model (orange), superimposed with the mutant MaAPX1^M36K^ mutant (cyan) (A). Structural insight of residue 36 (B). Table S1. Correlation analysis of H_2_O_2_ content and APX activity in banana peel with three different ripening characteristics. Table S2. The oligonucleotide sequence of MaAPX1 and the responding mutation site in MaAPX1 for RT-PCR and qRT-PCR.
