# Supplementary material for: Study on a Mechanism of Improving MaAPX1 Protein Activity by Mutating Methionine to Lysine
Source: Antioxidants (Basel). 2024 Jul 14;13(7):843. doi: 10.3390/antiox13070843 (PMC11273533; doi:10.3390/antiox13070843)

Table. S1 Correlation analysis of H_2O_2 content and APX activity in banana peel with three different ripening characteristics.

	H_2O_2 content of Control	H_2O_2 content of C_2H_4	H_2O_2 content of 1-MCP
Total APX activity of Control	0.749*		
Total APX activity of C_2H_4		0.904*	
Total APX activity of 1-MCP			0.955**

Note: ‘*’ indicates significant correlation, ‘**’ indicates extremely significant correlation

Table. S2 The oligonucleotide sequence of MaAPX1 and the responding mutation site in *MaAPX1* for RT-PCR and qRT-PCR. *MaAPX1*, *GSMUA_Achr5P07280_001 (XM_009401612)*；*MaActin-3*, *XM_009418623*.

Assay	Gene	Forward（5′-3′）	Reverse(5′-3′)
RT-PCR	*His-MaAPX1*	CCGCGCGGCAGCCATATGGCGAAGTCGTATCCGACGGTGA	AGTCATGCTAGCCATATGTTAAGCCTCAGCAAATCCGAGTTCT
	MaAPX1^{C32S}	ATCGCCGAGAAGAACAGTGCCCCGTTG	CAACGGGGCACTGTTCTTCTCGGCGAT
	MaAPX1^{M36K}	GACAAGCCTGAACCTCCCGT	GTCCAAGCCCCCTCAAAACC
qRT-PCR	*MaAPX1 (XM_009401612)*	CCGCGCGGCAGCCATATGGCGAAGTCGTATCCGACGGTGA	AGTCATGCTAGCCATATGTTAAGCCTCAGCAAATCCGAGTTCT
	MaActin-3 (XM_009418623)	TGGTATGGAAGCCGCTGGTA	TCTGCTGGAATGTGCTGAGG

Figure S1 Sequence alignment of APX proteins from various species. Comparison of the partial sequences of MaAPX1 with APX proteins of other plant species. The Cys32 and Met36 in MaAPX1 are highlighted by arrows.AtAPX1, NP_001030991.2 ascorbate peroxidase 1 [Arabidopsis thaliana]; NaAPX2, XP_019250347.1 PREDICTED: L-ascorbate peroxidase 2, cytosolic [Nicotiana attenuata]; OsAPX2, XP_015646556.1 L-ascorbate peroxidase 2, cytosolic [Oryza sativa Japonica Group]; SlAPX2, NP_001318094.1 L-ascorbate peroxidase 2, cytosolic [Solanum lycopersicum]; CpAPX2, XP_021911667.1 L-ascorbate peroxidase 2, cytosolic [Carica papaya]; ZaAPX, AAC08576.1 ascorbate peroxidase [Zantedeschia aethiopica].

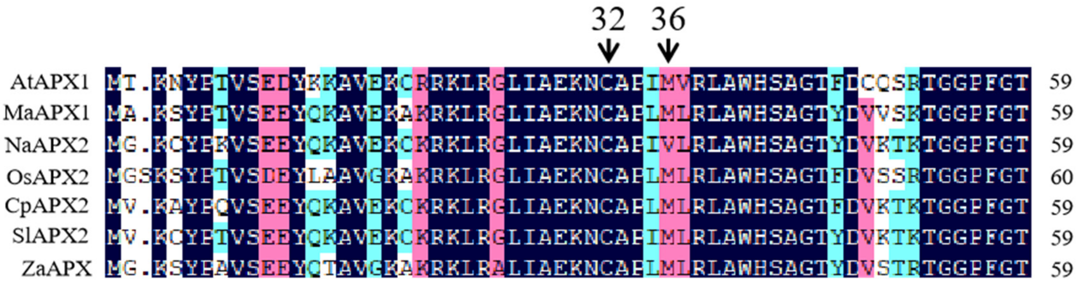

Figure S2 SDS-PAGE of purified expressed recombinant MaAPX1-His, MaAPX1^{M36K}-His and MaAPX1^{C32S}-His from *E. coli* BL21.

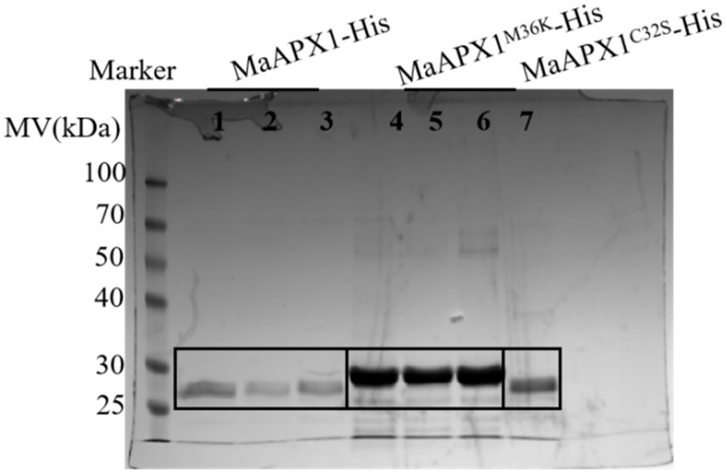

Figure S3 Structure of MaAPX1 predicted model (orange), superimposed with the mutant MaAPX1^{M36K} mutant (cyan) (A). Structural insight of residue 36 (B).

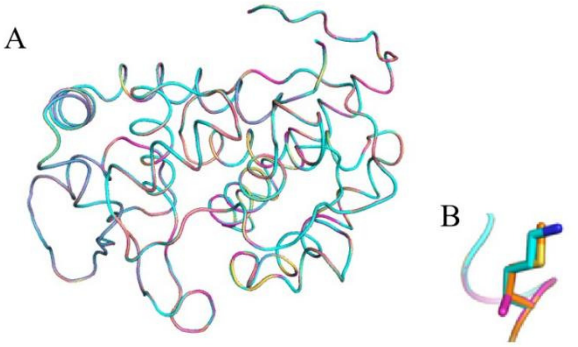

Supplement: Supplementary file 1 [file antioxidants-13-00843-s001.zip › antioxidants-3013798-supplementary.pdf]
